# A Comprehensive Sequence and Disease Correlation Analyses for the C-Terminal Region of CagA Protein of *Helicobacter pylori*

**DOI:** 10.1371/journal.pone.0007736

**Published:** 2009-11-06

**Authors:** Youlin Xia, Yoshio Yamaoka, Qi Zhu, Ivan Matha, Xiaolian Gao

**Affiliations:** 1 Department of Biology and Biochemistry, University of Houston, Houston, Texas, United States of America; 2 Department of Medicine-Gastroenterology, Veterans Affairs Medical Center and Baylor College of Medicine, Houston, Texas, United States of America; 3 Department of Environmental and Preventive Medicine, Oita University Faculty of Medicine, Yufu, Japan; University of Hyderabad, India

## Abstract

Chronic *Helicobacter pylori* infection is known to be associated with the development of peptic ulcer, gastric cancer and gastric lymphoma. Currently, the bacterial factors of *H. pylori* are reported to be important in the development of gastroduodenal diseases. CagA protein, encoded by the *cagA*, is the best studied virulence factor of *H. pylori*. The pathogenic CagA protein contains a highly polymorphic Glu-Pro-Ile-Tyr-Ala (EPIYA) repeat region in the C-terminal. This repeat region is reported to be involved in the pathogenesis of gastroduodenal diseases. The segments containing EPIYA motifs have been designated as segments A, B, C, and D; however the classification and disease relation are still unclear. This study used 560 unique CagA sequences containing 1,796 EPIYA motifs collected from public resources, including 274 Western and 286 East Asian strains with clinical data obtained from 433 entries. Fifteen types of EPIYA or EPIYA-like sequences are defined. In addition to four previously reported major segment types, several minor segment types (e.g., segment B′, B′′) and more than 30 sequence types (e.g., ABC, ABD) were defined using our classification method. We confirm that the sequences from Western and East Asian strains contain segment C and D, respectively. We also confirm that strains with two EPIYA segment C have a greater chance of developing gastric cancer than those with one segment C. Our results shed light on the relationships between the types of CagAs, the country of origin of each sequence type, and the frequency of gastric disease.

## Introduction

*Helicobacter pylori* is a Gram-negative bacterium etiologically involved in peptic ulcer disease, gastric adenocarcinoma, and primary gastric B-cell lymphoma [1]. Although infection with *H. pylori* almost always results in chronic active gastritis, only a fraction of those infected develop clinical disease. While this phenomenon remains unexplained, host genetics, host immune response, and the relationship of the host response to bacterial virulence factors are likely to be important factors. A tremendous number of groups have investigated the roles of putative virulence factors of *H. pylori*, and the best studied is the CagA protein [2]–[7]. CagA producing strains are reported to be associated with severe clinical outcomes, especially in Western countries [8]–[11].

**Figure 1:**
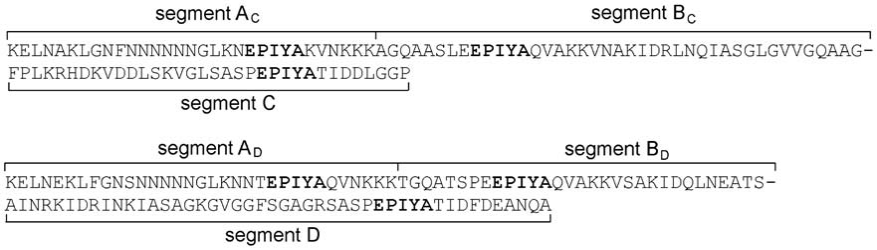
Definitions of segments around EPIYA motif (EPIYA or EPIYA-like sequences). The upper sequences are typical CagA sequences with Western type and the lower sequences are typical CagA sequences with East Asian type. Segments A, B, B′, and B′′have subscripts C and D, indicating that the sequences containing segments A, B, B′, and B′′ contain segments C and D, respectively. For example, the notation EPIYA-A_C_ signifies segment A from a CagA sequence containing the segment C.

CagA is a highly immunogenic protein with a molecular weight between 120 and 140 kDa [12], [13]. Variation in the size of CagA is due to the presence of a variable number of repeat sequences located in the 3′ region of the gene [12], [14]–[16]. The repeat regions contain the Glu-Pro-Ile-Tyr-Ala (EPIYA) motif. To characterize the different sequence patterns in the 3′ region, at least four methods of classification are typically reported. First, the terms D1, D2, and D3 are used to designate three specific sequences [12]. Second, sequences are denoted with combinations of R1, R2, and R3 [14], [15]. Third, each EPIYA motif is assigned a motif type (e.g., EPIYA-A, -B, -C, or –D motif) [17], [18], [19]. Finally, sequences are annotated according to segments (20–50 amino acids) flanking the EPIYA motifs (segments EPIYA-A, -B, -C, or –D) [20–23], after the identification of the essential CagA phosphorylation sites as confirmed by mutagenesis during infection and transfection [24]. Initially, the two Csk binding sites are designated as segments EPIYA-A and –B, and the Src homology 2 (SH2) domain of Src homology 2 phosphatase (SHP-2) binding sites in Western and East Asian type CagA are designated as segments EPIYA-C and –D, respectively. Here, “motif” and “segment” are used to designate the five-member sequence (EPIYA) and the short sequences around the EPIYA motif, respectively (Figure 1). However, none of the four sequence classification methods work well with non-standard sequences, and a modified classification method was deemed necessary.

CagA is encoded by the *cagA* gene, which is located at one end of the *cag* pathogenicity island (PAI) [25]. The *cag* PAI encodes a type IV secretion system, by which CagA proteins are delivered into host cells [26–30]. CagA interacts with various target molecules in addition to Csk and SHP-2, including Src [31], [32] and Abl [33]. Recent study clearly confirmed that almost one dozen of factors such as SHP-1, Grd2, Grb2, phosphatidylinositol 3-OH kinase (PI3K), have also binding abilities to CagA phosphorylation sites [34]. Mutations of SHP-2 have been found in various human malignancies and altered SHP-2 signaling culminates in the development of gastric adenocarcinoma in genetically engineered mice [35], [36], indicating that SHP-2 is involved in the development of gastric cancer. Recent studies reported that the East Asian type CagA containing segments EPIYA-D exhibits stronger binding activity for SHP-2 and a greater ability to induce morphological changes in epithelial cells than Western type CagA containing segments EPIYA-C [17], [20], [23]. The recent study also showed that *H. pylori* strains possessing East Asian type CagA have an ability to induce higher amounts of interleukin-8 from gastric epithelial cells than *H. pylori* strains possessing Western type CagA [37]. Accordingly, East Asian strains are believed to be more virulent than Western strains, and this might be the reason why the incidences of gastric cancer in East Asian countries are relatively higher than those in Europe, North America, and Australia (Data available at http://www-dep.iarc.fr/). In addition, the incidence of gastric cancer is reported to be higher in patients infected with strains carrying multiple EPIYA repeats compared to those infected with strains of a single repeat [14], [15], [38], [39].

However, there are also controversial reports that the genotypes (DNA analysis) of the CagA repeat region are not associated with clinical outcomes [40]–[43]. This controversy might be due in part to the fact that genotypes are not necessarily mutations in protein sequences and that the previous studies of the diversity of CagAs and the relationship of diseases and protein sequence types used only limited information, mostly relying on their own data sets. Indeed, there lacked comprehensive study considering all CagAs deposited in GenBank (http://www.ncbi.nlm.nih.gov/). Moreover, although CagA EPIYA repeats can be assigned to consensus sequence types, the existing sequence analyses did not completely consider the sequence variation patterns in the CagA repeat region. An in-depth analysis of the non-typical type repeats [15], [44] becomes necessary for addressing the question. In this study, we used sequence comparison and statistical method to analyze 560 unique CagAs selected from 4,534 CagAs from three data sources. Our results shed light on the relationships between the types of CagAs, the country of origin of each sequence type, and the frequency of gastric disease.

## Results and Discussion

### EPIYA Motifs Classification

By sequence alignment or pattern comparison, we found that there were sequences similar to EPIYA (such as EPIYT, ESIYT), although most sequences contained EPIYA. In this study, the EPIYA or EPIYA-like sequences were defined as any five member amino acid sequence with at least three amino acids corresponding to the sequence, EPIYA (where Y is always constant). By searching all sequences before data filtering, we obtained 16 types of EPIYA or EPIYA-like sequences. Of these, 15 types were chosen for further study because their surrounding sequences were similar to those of EPIYA (Table 1), indicating that these sequences might have a function similar to EPIYA. One sequence, MAIYA, from entry ABA26023 was excluded because the pattern of its flanking sequences was very different from those of the other 15 types of EPIYA or EPIYA-like sequences (Table 1). The 15 types listed in Table 1 are called EPIYA “motifs” for simplicity, in this work.

**Table 1:** Frequencies of the 15 types of EPIYA motifs.

Motif	EPIYA	EPIYT	ESIYA	ESIYT	EPIYV	EHIYA	ELIYA	EPVYA
Freq.	1657	92	24	7	3	2	2	2
Motif	EPIYD	EPIYS	EPKYA	EPRYA	ETIYA	KPIYA	NPIYA	Total
Freq.	1	1	1	1	1	1	1	1,796

The frequency of each EPIYA motif in the filtered data set is listed in Table 1. In total, 1,796 EPIYA motifs were obtained from the 560 CagAs. On average, each CagA sequence contained approximately three EPIYA motifs. The three most frequent EPIYA motifs were EPIYA (1,657/1,796 = 92.3%), EPIYT (92/1,796 = 5.1%), and ESIYA (24/1,796 = 1.3%).

### EPIYA Segments Classification

We categorized the EPIYA segments according to the segments flanking the EPIYA motifs (Figure 1). In addition to the four major segments originally designated, EPIYA-A, -B, -C, and –D [20], [22], we designated several minor segments, including EPIYA-B′ and -B′′. Representative examples of these types of segments, derived from the 560 CagAs, are listed in Table 2 (a few more other types of segments with frequency less than 10 are given in Table S1. For simplicity, we refer to segment EPIYA-A, -B, -C, or –D as segment A, B, C, or D. Segments A, B, B′, and B′′ have subscripts C and D, which indicate that the sequences that contain segments A, B, B′, and B′′ contain segments C and D, respectively (Figure 1). However, 19 short sequences did not contain either segments C or D, and we manually assigned a subscript C or D to the segment type, according to their sequence patterns.

**Table 2:** Representative segments of EPIYA motifs^a^.

Type	Freq.	Representative sequence
A_C_	272	KELNAKLGNFNNNNNNGLKN..EPIYAKVNKKK
A_D_	295	KELNEKLFGNSNNNNNGLKNNTEPIYAQVNKKK
B_C_	262	TGQVASPEEPIYAQVAKKVNAKIDRLNQIASGLGGVGQAAG
B_D_	281	TGQATSPEEPIYAQVAKKVSAKIDQLNEATS
C	343	FPLKRHDKVDDLSKVGRSVSPEPIYATIDDLGGP
D	284	AINRKIDRINKIASAGKGVGGFSGAGRSASPEPIYATIDFDEAN
B′_C_	10	AGQAASPEEPIYAKVNKKK
B′_D_	14	AGQATSPEEPIYAQVNKKK
B′′_D_	19	AINRKIDRINKIASAGKGVGGFSGAGRSANPEPIYAQVARKVSA-KIDQLNEATS
Total	1,780	

a Note: the values in the table are the frequencies of similar sequences, not the number of identical sequences within a sequence type. Other segments of 16 EPIYA motifs are listed in Table S1.

We named the minor segments according to the patterns of the sections immediately following EPIYA (Table 2). This was because the four amino acids, TIDD and TIDF, following EPIYA in segments C and D, respectively are reported to be important for the binding of SHP-2 [17], [24]. For example, segments B′_C_ and B′_D_ are shorter versions of segments B_C_ and B_D_, respectively (Table 2). In segment B′′_D_, the sequences before EPIYA are similar to those of segment D, whereas the sequences after EPIYA are similar to those of segment B_D_.

The segment B displayed the biggest change in the five amino acids; EPIYA motif (Table S2). For the three most frequent motifs (excluding EPIYA), 89 out of 92 EPIYTs, all 23 ESIYAs, and all 7 ESIYTs, appear in segment B. Interestingly, 88 EPIYT motifs belong to the segment B_C_, and only 1 EPIYT belongs to the segment B_D_. In contrast, the changes of the five amino acids in segments A, C, and D were relatively small. In other reports [18], [19], the NPIYA, EPIYT, ESIYA and ESIYT motifs were named as A′, B′, B″ and B″′, respectively. However, their terminology seems to be confusing, otherwise all 15 types of pseudo EPIYA motifs should have different names. Their motif A′ belonged to our segment A and their B′, B″, and B″′ fell into our segments B, B′, or B′′ (Table S2).

### CagA Sequence Type Classification

Each CagA sequence was assigned a sequence type consisting of the names of the EPIYA segments in its sequence (such as ABC or ABD) (Table S3). Depending on the number of EPIYA segments, they are termed as AnBnCn or AnBnDn, where “n” is the repeating motifs and does not have to be equal for A, B, C, and D types (e.g., ABCCCC). In the event that there was an additional segment that lacked an EPIYA motif between two neighboring EPIYA segments, a hyphen was added between the two EPIYA segments (e.g., A-C, A-D). In total there were 28 segments without EPIYA motifs between two neighboring EPIYA segments among the 560 CagAs (Table S3). These 28 interval segments are of various lengths and contents. In total, 41 different sequence types were found (Table S4). Among the 41 sequence types, 32 sequence types are remained (Table 3) after removing the types containing rare EPIYA segment types (i.e., B′′_C_, C′, D′, C′′ and D′′). The majority of the sequences were of types ABD (43%) and ABC (30%). Interestingly, there were no CagA sequences containing both segments C and D. This suggests hybridization (recombination) between Western and East Asian CagA is very rare.

**Table 3:** Frequencies of the 32 sequence types^a^.

Seq. Type	Freq.	Seq. Type	Freq.	Seq. Type	Freq.	Seq. Type	Freq.
ABD	240	AB′-ABD	4	C	2	ABCCCC	1
ABC	167	A-D	4	A	1	A-B″D	1
ABCC	51	A-ABD	3	AB′B′BC	1	AB-D	1
ABB″D	16	AB-ABD	2	ABB″BD	1	ABD-ABD	1
AB	15	AB′B′BD	2	AB′BCC	1	ABD-BD	1
ABCCC	10	AB′BD	2	AB′-C	1	ABD-D	1
AB′BC	6	ABCCCCC	2	AB-C	1	A-CCC	1
A-C	5	AB′D	2	ABCB″CC	1	CC	1

a All sequence types are listed in Table S3. Other sequence types are listed in Table S4.

A small number of CagAs were classified differently between our current study and previous studies (examples shown in Table S5. For example, the CagA sequence of BAF45291 was classified as AC in a previous study [44]. However the sequence type was A-C in our classification, which meant that an interval segment (VKAKIDQLNQAASGFGNVGQAG) lacking EPIYA-like motif was present between the sequences A and C. For the CagA sequence of BAF45283, the sequence type was reported to be ABDD in a previous study [44]. However, the sequence type was classified as ABB″D in this work. The 3^rd^ segment that differs between the two studies (D vs. B″) is AINRKIDRINKIASAGKGVGGFSGAGRSASP**EPIYA**QVAKKVSAKIDQLNEATS. In this segment, the part before the EPIYA motif is similar to segment D, whereas the part after the EPIYA motif is similar to segment B. Obviously, this segment is neither D nor B, rather B″, a variant of segment B (Table 2). Overall, we believe that the definitions of segment and the sequence classifications used in this study are more meaningful and accurate than those used in previous studies.

Each of the 560 CagAs was found to have at least one, and as many as seven, EPIYA segments (or EPIYA motifs). The distributions are 3, 27, 416, 86, 23, 3, 2, and 0 for number of sequences containing 1 through 8 EPIYA segments (Table S6), respectively. For example, a sequence of type A has only one EPIYA segment A and a sequence of type ABCCCCC has seven EPIYA segment, including five repeats of segment C. The majority (74% = 416/560) of sequences had three EPIYA segments.

### Detailed Analyses of EPIYA Segments

The EPIYA segment types were defined according to the segment patterns (Table 2); however the composite amino acids varied slightly within each segment type. The two most frequent segments in segments A, B, C and D are shown in Table 4. The segments of EPIYA-A_C_ or -A_D_ contain from two to eight Ns (Gln) at the upstream of the pseudo EPIYA-A_C_ or -A_D_ motif. The segments C and D have higher consensus than segments A_C_, A_D_, B_C_ and B_D_.

**Table 4:** Two most frequent EPIYA segments^a^.

	Segment	Ratio
A_C_	KELNAKLGNFNNNNNNGLKN..EPIYAKVNKKK	53/272
A_C_	KELNAKLGNFNNNNNNGLKNSTEPIYAKVNKKK	22/272
A_D_	KELNEKLFGNSNNNNNGLKNNTEPIYAQVNKKK	53/272
A_D_	XXXXXKLFGNSNNNNNGLKNNTEPIYAQVNKKK	22/272
B_C_	TGQVASPEEPIYAQVAKKVNAKIDRLNQIASGLGGVGQAAG	25/262
B_C_	AGQAASPEEPIYAQVAKKVNAKIDRLNQIASGLGGVGQAAG	19/262
B_D_	TGQATSPEEPIYAQVAKKVSAKIDQLNEATS	25/262
B_D_	TGQVASPEEPIYAQVAKKVSAKIDQLNEATS	19/262
C	FPLKRHDKVDDLSKVGRSVSPEPIYATIDDLGGP	144/343
C	FPLKRHDKVDDLSKVGRAVSPEPIYATIDDLGGP	50/343
D	AINRKIDRINKIASAGKGVGGFSGAGRSASPEPIYATIDFDEAN	144/343
D	AINRKIDRINKIASAGKGVGGFSGAGRSASPEPIYATIDFDETN	50/343

a X represents unknown amino acids; the amino acids which are different in two sequences shown are highlighted; Ratio  =  (Frequency of the type)/(Total frequency).

**Figure 2:**
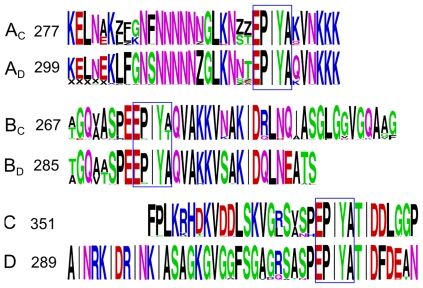
WebLogos of aligned segments of EPIYA-A, -B, and -C/D. The numbers of sequences for each WebLogo are indicated. The sequences were aligned using BioEdit. Z represents space inserted by BioEdit and X represents unknown amino acids.

There were obvious differences between segment C and D when analyzed using the program, WebLogo (Figure 2). The segments were aligned using BioEdit before they were entered into WebLogo. As WebLogo had a problem analyzing a column of aligned sequences if BioEdit had added many spaces, all spaces in the sequence alignments were replaced by Z (meaning zero or nothing). In this way, the inserted space (Z) and the minor amino acids were easily identified. In the alignments, X indicates that an amino acid was not-available. As shown in Figure 2, the lengths of segments A_C_ and A_D_ are the same and the sequences of segments A_C_ and A_D_ are very similar. However the lengths of segments B_C_ and B_D_, and the segments C and D are quite different. The sequences after the stretch of amino acids, QVAKKV, in segments B_C_ and B_D_ were highly variable, while the sequences of segments C and D were completely different. Overall, the sequence main variation between Western and East Asian strains starts after QVAKKV in segments B_C_ and B_D_.

The four amino acids TIDD and TIDF following EPIYA motifs in segments C and D are reported to be important for the binding SHP-2 [17], [24]; therefore, the frequency of the four amino acids following EPIYA motifs in all EPIYA segments may be useful. As illustrated in Table S7, the sequences, KVNK and QVNK, occupy this position in the majority of segments A_C_ and A_D_, respectively. QVAK occupied this position in most segments B_C_ and B_D_. In the literature [17], the criteria for identifying EPIYA segments C and D are that the EPIYA motif is followed by TIDD and TIDF, respectively. However, by sequence pattern comparison, we found that EPIYA also belongs to segment C if it is followed by TIEE, TIDE, SIDD, TIDG, TIAE, or TIAD. If EPIYA is followed by TIDS, then it belongs to motif type D. As shown in Table S2, the segments B, B′, and B′′ had the biggest changes in their composite five amino acids. However, the four amino acids following the EPIYA motif were most variable in segment A (Table S7).

### Correlation of Sequence Types and Geographic Areas

*H. pylori* strains from different geographic areas are associated with clear phylogeographic differentiation and *H. pylori* populations tend to spread along the lines of human migratory fluxes [45]–[50]. Furthermore, several studies concluded that CagA isoforms with segments C and D are related to Western and East Asian countries, respectively [14]–[16]. We tested this hypothesis using our comprehensive system of CagA classification. The frequency of each sequence class in individual countries is shown in Table 5. As expected, all 227 (100%) samples from Western countries contain EPIYA segment C. In contrast, of 307 sequences from East Asian countries (Japan, China, Korean, and Viet Nam), 26 (∼8%) contain EPIYA segment C instead of segment D. Interestingly, of the 21 Japanese strains with CagA sequence types related to segment C, 17 have names beginning with OK (Table S8), signifying that they were isolated in Okinawa, Japan (discussed below). The prevalence of sequences containing segments C and D in Southeast Asian countries (Thailand and Malaysia) were the same; and all samples from Iran, Kazakhstan (Kazak), and India were classified as segment C, although they are Asian countries. Overall, we found that it is largely true that CagA with sequences segments C and D are related to Western and East Asian countries, respectively; however, there are some exceptions for East Asian strains. Southeast Asian countries form the geographical border between segment C and segment D. The fact that some East Asian countries have Western type CagA reflects the partial transmission of *H. pylori* from Western to East Asian countries either during the human migration long time ago or recent transmission.

**Table 5:** Frequency of CagAs with respect to country^a^.

Country	total #	# of seq. containing EPIYA-C	# of seq. containing EPIYA-D
Japan	249	21	228
China	48	4	44
Korea	6	1	5
Viet Nam	4	0	4
Thailand	5	2	3
Malaysia	3	2	1
Iran	5	5	0
India	4	4	0
Kazakhstan	3	3	0
Greece	100	100	0
Italy	34	34	0
Sweden	5	5	0
Ireland	3	3	0
USA	22	22	0
Costa Rica	33	33	0
Colombia	24	24	0

a Austria, Chile, and Germany each have one strain. The country information of 11 sequences or strains is not available.

As mentioned above, there are 21 strains from Japan with sequences related to EPIYA segment C instead of segment D (Table 5). The detailed information of these 21 strains is given in Table S8. Most of these segment C strains were isolated from Okinawa, which was governed by the United States from the end of World War II until 1972, and even today there are many US populations living in Okinawa. These data show that transmission of *H. pylori* between different populations may not be a rare event. In fact, previous reports of native Americans in Peru show that all *H. pylori* strains in this population are of the Western type [51], while only 4 of 17 strains isolated from American primitive, an isolated group living in the Amazonian jungles of Colombia, were East Asian type strains [48]. Based on our data, the Western strains are more easily transferred to East Asian people than the other way around. Another possibility for Western type CagA in Okinawa is that the Okinawan CagA is the novel type CagA; the origin did not come from modern Western people, but came to Japan long ago. Further studies will be necessary to test this hypothesis. If it proves true, elucidating the mechanism will be important for understanding the transmission of *H. pylori* in human populations.

Among the 21 strains from Okinawa, 20 contain EPIYA segment B (Table S8). Of 20 EPIYA motifs in segment B, 15 are EPIYT and 4 are ESIYT. Comparing this information with the data in Table S2, we found that the frequencies of the EPIYT and ESIYT motifs among the sequences of the 21 Okinawa strains are also relatively high. Detailed analyses for large number of strains from Okinawa will provide us some information about the roles and evolution of EPIYA motifs.

### Correlation of Sequence Types and Strain Diseases

We were able to obtain clinical information for 433 strains out of the 560 strains in our data set (Table 6). In our data sheet, disease G contains gastritis, atrophic gastritis, epigastrial pain, gastric hyperplastic polyp, non-ulcer dyspepsia, chronic gastritis, chronic atrophic gastritis, and chronic gastritis-associated dyspepsia as well as “gastris”, which are regarded as typo of gastritis. Disease DU and GU (peptic ulcer PU  =  DU + GU) represent duodenal ulcer and gastric ulcer, respectively. Disease GC contains gastric cancer, gastric carcinoma, gastric adenocarcinoma, gastric adenoma and adenomatous polyps. Disease MALT contains MALT lymphoma and MALToma. Disease E represents esophagitis. Among those 433 samples, 42%, 32%, and 20% of the patients had diseases G, PU, and GC, respectively, which shows that there is a potential for selection bias in the sequence samples. For example, the prevalence of GC is approximately 3% in *H. pylori*-positive patients [52]. Nonetheless, the data are useful when comparing patterns of sequence types among diseases.

**Table 6:** Frequency and percentage of strains of certain type disease^a^.

Disease	G	DU	GU	GC	E	MALT	Total
Occurrence	181	90	43	87	21	5	433
Percentage	42%	21%	10%	20%	5%	1%	100%

a The diseases are designated in the text.

We compared three types ABC, ABD and ABCC in relation to clinical outcomes. Other EPIYA types were excluded since the number of other minor types was relatively small. As shown in Table 7, the prevalence of ABCC was 22% (17/[22 + 38 + 17]) in GC; whereas only 12% (18/[65 + 66 + 18]) in G and 7% (8/[42 + 64 + 8]) in PU. The ratio of ABCC/ABC was therefore significantly higher in GC (17/22 = 0.77) than in PU (8/42 = 0.19) and G (18/65 = 0.28) (The calculated chi-square is 8.24 and 6.22, and the probabilities of null hypothesis are less than 0.03 and 0.01, respectively). The data that strains with more EPIYA segment C have a greater chance of developing gastric cancer is consistent with previous studies [15], [38]. The ratio of ABD/ABC was also higher in GC (38/22 = 1.73) than in PU (64/42 = 1.52) and G (66/65 = 1.02); however the differences were not statistically significant (The calculated chi-square is 0.14 and 2.79, and the probabilities of null hypothesis are more than 0.90 and 0.10, respectively).

**Table 7:** EPIYA types and clinical outcomes^a^.

	Total	G	PU	GC
ABC	129	65, 50%, 1.0	42, 33%, 1.0	22, 17%, 1.0
ABD	168	66, 39%, 0.8	64, 38%, 1.2	38, 23%, 1.3
ABCC	43	18, 42%, 0.8	8, 19%, 0.6	17, 40%, 2.4

a PU  =  DU + GU. Other diseases are designated in the text. The strains with unavailable disease information are not included.

The 145, 44, and 169 sequences of types ABC, ABD, and ABCC, respectively, from strains with disease information were used for phylogenic analysis with ClustalW (http://align.genome. jp/). The resulting trees are shown in Table S9, S10 and S11 in the supplementary material. The phylogenetic analysis did not reveal any association between a particular disease and a specific CagA sequence.

### Conclusion

In this study, 560 unique CagA sequences containing EPIYA-like motifs were analyzed and in addition to the four previously reported major CagA segment types (A, B, C and D), we found that there are various novel types. Our results allow a clearer classification of the CagA protein sequences and provide a basis for further molecular studies of the pathogenicity of this important protein. In addition, we confirmed that strains with two EPIYA segment C have a greater chance of developing gastric cancer than those with one segment C. However, we did not find any association between a particular disease and specific CagA sequences through phylogenic tree analysis and further studies with larger number of sequences might be necessary whether the specific CagA sequences are involved in the development of clinical outcomes.

## Materials and Methods

### Data Collection

Three databases, NCBI (National Center for Biotechnology Information, U.S. National Library of Medicine, www.ncbi.nlm.nih.gov), UniProtKB/Swiss-Prot (the Swiss Institute for Bioinformatics and the European Bioinformatics Institute, www.ebi.ac.uk/swissprot/), and DDBJ (DNA Data Bank of Japan, the National Institute of Genetics, www.ddbj.nig.ac. jp/), were used to obtain CagA sequencing data. As of Apr 16, 2007, 1,423 entries were retrieved by searching “protein” at NCBI for “Helicobacter pylori CagA” with display format of “GenPept (Full)”. All related data were saved to a local disk. 1,034 entries were retrieved by searching the library, “UniProtKB/Swiss-Prot & UniProtKB/TrEMBL” at Swiss-Prot for “Helicobacter pylori CagA”. The related data were downloaded in a “Flat File Format”. Similarly, 2,077 entries were retrieved by searching “protein” at DDBJ for “Helicobacter pylori CagA”. By choosing “Complete entries”, the data were saved as ASCII text on a local disk. The data from DDBJ include the data from NCBI and UniProtKB/Swiss-Prot. We found that the sequences from NCBI included all sequences from UniProtKB/Swiss-Prot and DDBJ; therefore, only the NCBI data were used for sequence analyses. We have collected clinical information for 433 strains related to *H. pylori* CagA. The information is from our data base (from Y.Y.), the NCBI database, and the literature [53], [54], [18], [19].

### Data Filtering

EPIYA motifs are located in the C-terminus of the CagA protein. 1,423 entries annotated as CagA in NCBI were downloaded from GenBank. Two rounds of data filtering were used to refine the data obtained from NCBI: (1) removing 832 sequences not containing EPIYA or EPIYA-like motifs (Table S12) and (2) removing 31 redundant sequences (Table S13). Among the 31 sequences, 18 sequences are completely same as others and 13 sequences are parts of others. After the two rounds of filtering, 560 unique CagAs containing EPIYA or EPIYA-like motifs remained (Table S3).

### Statistical Analyses

Chi-square test is used to test the statistical significance of the difference of strains of sequence types ABCC and ABC in disease groups GC, PU and G. From Table 7, 17 and 22 strains with ABCC and ABC types appear in disease GC group, and 8 and 42 strains with ABCC and ABC types appear in disease PU group. The calculated chi-square (http://math.hws.edu/javamath/ryan/ ChiSquare.html ) is 8.24 from a 2×2 matrix. Similarly, 17 and 22 strains with ABCC and ABC types appear in disease GC group, and 18 and 65 strains with ABCC and ABC types appear in disease G group. The calculated chi-square is 6.22 from a 2×2 matrix. Then from a chi-square table, the probabilities of null hypothesis are less than 0.03 and 0.01, respectively, with a df  =  1 (df: degree of freedom).

### Software for Data Analysis

Home-made program based on MATLAB was used to extract information from the original data retrieved from NCBI, search the sequences, sort the sequences according to disease, create files in FASTA format, etc. BioEdit and WebLogo were used to align and display protein sequences [55], [56]. ClustalW (http://align.genome.jp/) and TreeView (http://taxonomy.zoology.gla.ac.uk/rod/treeview. html) were applied to build and view phylogenic trees.

## Supporting Information

Table S1 Full list of representative segments of EPIYA motifs Note: the values in the table are the frequencies of similar sequences, not the number of identical type sequences within a sequence. The highlighted segments are removed in Table 2.
(0.02 MB XLS)

Table S2 Distribution of EPIYA motifs in segments A, B, C and D
(0.02 MB XLS)

Table S3 Unique CagA sequences and their sequence types
(0.10 MB XLS)

Tabld S4 Frequencies of all sequence types All sequence types are listed in Table 3S in supplementary.pdf. The highlighted sequence types are removed in Table 3.
(0.02 MB XLS)

Table S5 Comparison of sequence classifications in literatures [1] T.Uchida, R. Kanada, Y. Tsukamoto, N. Hijiya, K. Matsuura, S. et al., Cancer Sci. 98 (2007) 521–528. [2] M. Naito, T. Yamazaki, R. Tsutsumi, H. Higashi, K. Onoe, et al., Gastroenterology 130 (2006) 1181–1190.
(0.02 MB XLS)

Table S6 Distribution of multiple repeats of EPIYA segments
(0.02 MB XLS)

Table S7 Distribution of the first four amino acids following EPIYA motifs
(0.02 MB XLS)

Table S8 Samples (from Japan) related to EPIYA-C
(0.02 MB XLS)

Table S9 The phylogenic tree of fragments ABC
(0.40 MB XLS)

Table S10 The phylogenic tree of fragments ABCC
(0.11 MB XLS)

Table S11 The phylogenic tree of fragments ABCC
(0.40 MB XLS)

Table S12 The information of sequences without EPIYA-like motif
(0.13 MB XLS)

Table S13 The information of redundant sequences *The sequences under ANo2 are completely same as or cover that under Ano. **Length2 are the length of sequences under ANo2.
(0.02 MB XLS)
