# Correction: A Comprehensive Sequence and Disease Correlation Analyses for the C-Terminal Region of CagA Protein of *Helicobacter pylori*

**DOI:** 10.1371/annotation/a15728ad-c776-49e4-b39d-bb134c16345c

**Published:** 2010-07-12

**Authors:** Youlin Xia, Yoshio Yamaoka, Qi Zhu, Ivan Matha, Xiaolian Gao

The NCBI protein accession numbers for the sequences employed in the analysis, and the details of the publications describing each sequence, were omitted from the supplementary information in the published article. The authors would like to apologize for this omission and would like to replace the supplementary Table S3 by the one provided here which includes this information: 

The authors would also like to acknowledge the work carried out by the different groups that described the sequences included in this study, and we have therefore included their relevant details in the revised table.

